# On different approximations to multilocus identity-by-descent calculations and the resulting power of variance component-based linkage analysis

**DOI:** 10.1186/1471-2156-4-S1-S72

**Published:** 2003-12-31

**Authors:** Harald HH Göring, Jeff T Williams, Thomas D Dyer, John Blangero

**Affiliations:** 1 Department of Genetics, Southwest Foundation for Biomedical Research, San Antonio, Texas, USA

## Abstract

An empirical comparison between three different methods for estimation of pair-wise identity-by-descent (IBD) sharing at marker loci was conducted in order to quantify the resulting differences in power and localization precision in variance components-based linkage analysis. On the examined simulated, error-free data set, it was found that an increase in accuracy of allele sharing calculation resulted in an increase in power to detect linkage. Linkage analysis based on approximate multi-marker IBD matrices computed by a Markov chain Monte Carlo approach was much more powerful than linkage analysis based on exact single-marker IBD probabilities. A "multiple two-point" approximation to true "multipoint" IBD computation was found to be roughly intermediate in power. Both multi-marker approaches were similar to each other in accuracy of localization of the quantitative trait locus and far superior to the single-marker approach. The overall conclusions of this study with respect to power are expected to also hold for different data structures and situations, even though the degree of superiority of one approach over another depends on the specific circumstances. It should be kept in mind, however, that an increase in computational accuracy is expected to go hand in hand with a decrease in robustness to various sources of errors.

## Background

All methods of statistical gene mapping by means of linkage and/or linkage disequilibrium use, in one way or another, the information on polymorphic phenotypesÂ-typically, the genotypes at one or several polymorphic marker lociÂ-to trace the inheritance of any specific chromosomal position through the available pedigree data. In variance-component (VC) linkage analysis, this transmission pattern is captured by an identity-by-descent (IBD) matrix, which contains the estimated proportions of alleles shared at a particular genomic location for all pairs of pedigree members. Normally, the observed marker locus genotypes provide only partial information about the meiotic transmissions of a given point on a chromosome, such that many different inheritance patterns are compatible with the observed marker locus genotypes. For reasons of computational simplicity, it is currently standard, though likely sub-optimal, practice in VC-based linkage analysis to form a weighted average of IBD sharing over all admissible segregation patterns, with the probability of each possible transmission pattern used for weighting. The resulting estimated IBD matrix is part of the variance-covariance matrix used to compute the likelihood on the data under an assumed multivariate normal distribution [1] or a multivariate *t *distribution [2].

The IBD matrix may be estimated from the genotype information at single marker loci, one locus at a time. Alternatively, the genotypes observed at several linked marker loci may be used jointly to infer the transmission pattern in the data set. Because the genotypes at a single marker locus are almost never fully informative, and because the joint use of several marker loci generally allows more information on the point-wise transmission pattern to be extracted, the "multi-point" approach is often preferred to the "two-point" approach. This is especially true for VC-based linkage analysis where, in contrast to penetrance-model-based linkage analysis, single-marker and multi-marker analyses are equally robust to misspecification of the trait phenotype-trait locus genotype relationship, for reasons explained by Göring and Terwilliger [3]. It should be kept in mind, however, that a multi-marker approach is not penalty-free even for VC-based linkage analysis, because multi-marker analysis is generally less robust to errors in pedigree structure and marker information [e.g., [4,5]].

A key problem with multi-marker analysis is its computational burden. The Elston-Stewart algorithm [6] allows likelihood computations on large pedigrees but only for a single marker locus or a small number of loci at most, and the Lander-Green algorithm [7] makes possible the joint analysis of many loci but only on pedigrees of moderate size. Several approximate approaches have been developed to overcome these limitations. Markov chain Monte Carlo (MCMC) methods [e.g., [8,9]] extend the feasibility of linkage analysis with regards to the complexity of a pedigree that can be handled while leaving it intact, and to the number of loci that can be analyzed jointly, by sampling from the permissible inheritance patterns. However, even these approaches can require long computation times. Furthermore, it is typically not clear how closely the obtained information on chromosomal transmissions approximates the information from an exact analysis. An alternative concept to approximating exact multi-locus analysis is sometimes referred to as multiple two-point analysis. The idea behind this approach is to combine the computational simplicity of single-marker analysis and the increased power of multi-marker analysis. In VC-based linkage analysis, this is achieved by first computing exact single-marker IBD matrices for all linked marker loci individually and by then combining these IBD matrices into an approximate multi-marker IBD matrix for a given chromosomal location [10,11].

Here, we describe an empirical power comparison between VC-based linkage analysis using single-marker (two-point) analysis, approximate multi-marker analysis using a multiple two-point approach, and approximate multi-marker analysis using a multipoint MCMC approach, to quantify the relative gain in power by increasing the computational complexity of IBD matrix estimation.

## Methods

### Data

The simulated data prepared for the Genetic Analysis Workshop (GAW) 13 were used for analysis. The data set comprises 4692 individuals in 330 pedigrees in total, modeled after the Framingham Heart Study [12]. The data set was "randomly" ascertained, i.e., without regard to a specific phenotype. The phenotypic and genotypic data from Cohort 2 was used, which consists of 1634 individuals of younger generations. Cohort 1 contains older individuals connecting the younger individuals together into larger pedigrees. No phenotypic or genotypic information from Cohort 1 was used here. Thus, for the most part, data were available only from the youngest one or two generations.

We analyzed height measured at the first clinic visit of this cohort (phenotype hgt1). This phenotype is largely controlled by additive genetic effects, which together explain 84% of the sex-specific variance. The most important quantitative trait locus (QTL), G_b1_, is located on chromosome 5 at 80.41 cM of the sex-averaged map and explains 40% of the sex-specific variance. The QTL is flanked by the eight marker loci c5g9-c5g16 (four on either side), which have roughly 10 cM inter-marker spacing. The observed genotypes at these eight marker loci were used for analysis. To better highlight the difference in power between VC-based linkage analysis based on the various examined approaches to IBD matrix estimation, two of these marker loci (c5g12 and c5g14) were made diallelic by combining all even and all odd alleles. The other six marker loci had stated heterozygosities of at least 0.68. Replicates 1–10 were analyzed. The simulation settings (i.e., the "answers") were known prior to analysis.

### Statistical analysis

Single-marker and various multi-marker VC-based linkage analyses were performed using eight linked marker loci. A sex-averaged map was used throughout, and absence of recombination interference was assumed in the analysis. Marker allele frequencies were estimated by a simple allele-counting algorithm on all genotyped individuals, regardless of relationship. Single marker IBD matrices were computed by computer program SOLAR version 1.7.3 [11], which used computer program FASTLINK version 3.0P [13] as the underlying computation engine for these IBD calculations. SOLAR's built-in multiple two-point regression-based approach [11] was used to combine the single-marker IBD matrices into approximate multi-marker IBD matrices. The computer program SIMWALK2 version 2.82 [8], which uses a MCMC approximation to exact likelihood computation, was used to compute true multi-marker IBD matrices. Standard VC-based linkage analysis was performed with SOLAR assuming phenotypic multivariate normality and using sex as a fixed effect covariate, based on the IBD matrices obtained by any of the three alternatives in turn.

## Results

### Power

Table 1 shows the maximum LOD score in the region around the QTL for the three different methods of IBD sharing computation for Replicates 1–10. In 9 out of 10 replicates, the maximum LOD score for the multiple two-point approach, which uses a regression procedure to combine the individual single marker IBD matrices into approximate multi-marker IBD matrices, is higher than the maximum LOD score obtained in two-point analysis, which is based on IBD matrices computed from the genotypes at single marker loci individually. The difference in magnitude between the two LOD score peaks is often quite substantial. The only replicate where the two-point approach is more powerful is the replicate giving the lowest LOD score peak for both methods.

The true multi-marker approach, using an MCMC approximation to compute multi-locus IBD probabilities, is in turn more powerful than the multiple two-point approach in 9 out of 10 replicates, in many cases giving a substantially higher LOD score peak. On average, the regression-based multiple two-point approach gives maximum LOD scores that are roughly intermediate between those from a single-marker and a true multi-marker approach.

### Localization

Table 2 shows the genetic distance between the chromosomal position where the maximum LOD score occurred and the true chromosomal position of the QTL for the different approaches to IBD sharing estimation for the same 10 replicates. The single-marker method fared poorly in comparison to the multi-marker approaches, giving much greater genetic distances on average. This was expected, because the two flanking marker loci were ~6 and ~12 cM away from the QTL, respectively. The regression-based multiple two-point approach and the MCMC-based multipoint approaches were used to compute IBD matrices every centimorgan (cM) and were comparable in accuracy of QTL localization.

## Discussion

### Differences in power

We have compared three different approximations to multi-marker IBD sharing computation with regards to power of VC-based linkage analysis. On the examined data, it is clear that the multipoint approach is more powerful than the multiple two-point approach, which in turn in more powerful than the two-point approach. In this data set, the multiple two-point method is able to capture more information on the chromosomal segregation pattern than a two-point approach, without a significant increase in computational burden. On the other hand, the multiple two-point approach clearly does not use all available information on the chromosomal transmissions among pedigree members contained in the observed genotypes.

The difference in power between the two considered multi-marker approaches is expected to be especially pronounced when the marker loci individually are quite uninformative (data not shown). The degree to which the true multipoint approach is preferred may scale differently depending on the reasons why individual marker loci provide little information, such as low heterozygosity, e.g., when single nucleotide polymorphisms are used, or when genotyped individuals are separated by multiple generations of ungenotyped individuals. The marker locus density is also expected to play a role.

We were unable to compute exact multi-marker IBD sharing probabilities on this data set for comparison because the pedigrees were found to be too large for such calculations, at least for the time being. We suspect that such an approach would be at least as powerful as the employed MCMC approximation, unless the sampler underlying the SIMWALK2 computer program biases the IBD sharing probabilities in a systematic fashion relative to the analyzed phenotype, which seems unlikely in this case given the "random" ascertainment of these pedigrees.

### Generality of findings

This has been an empirical investigation on a data set with specific characteristics of the pedigrees, the phenotype, and the marker loci and their genotypes. While we suspect that our overall conclusion, that power to detect linkage increases with an increased computational sophistication in computing IBD sharing probabilities, holds more generally, the following caveats should be kept in mind.

The data were simulated to be without any errors. While the simulations were based on sex-specific recombination fractions, the analysis assumed equal genetic distances for both sexes. (This choice was made to keep the conditions as similar as possible between the different IBD calculations. While SIMWALK2 can handle sex-specific maps currently, SOLAR's multiple two-point approach cannot at present.) However, besides this one source of error, the data and analyses represent an ideal situation that is unrealistic for real data. It is known, however, that multi-marker analysis is generally less robust to errors in pedigree structure, genetic marker map, marker allele/haplotype frequencies, and marker genotypes [e.g., [4,5]]. We suspect that the multiple two-point approximation is more robust to most if not all of these errors than true multipoint analysis. Thus, there is a trade-off between increasing accuracy of computation and resulting increase in power on the one hand and robustness to errors on the other hand. The critical point of balance between both considerations likely falls on different error levels for different data sets and conditions.

## Figures and Tables

**Table 1:** Maximum LOD scores for three different methods of IBD sharing estimation

**Replicate**	**Two-Point**	**Multiple Two-Point (Regression)**	**Multipoint (MCMC)**
1	3.54	6.08	7.18
2	4.48	5.23	7.44
3	2.29	2.83	4.70
4	3.30	5.64	6.41
5	4.78	5.05	6.64
6	4.37	6.50	6.52
7	4.21	7.18	7.03
8	3.28	4.36	5.12
9	3.03	3.70	4.44
10	1.35	0.74	1.76
Mean	3.46	4.73	5.72
Median	3.42	5.14	6.47

**Table 2:** Distances between positions of maximum LOD scores and QTL^A^

**Replicate**	**Two-Point**	**Multiple Two-Point (Regression)**	**Multipoint (MCMC)**
1	17.0	5.5	4.5
2	17.0	1.5	0.5
3	6.2	4.5	5.5
4	6.2	1.5	4.5
5	17.0	15.5	9.5
6	17.0	14.5	8.0
7	11.9	0.5	0.5
8	11.9	2.5	5.5
9	6.2	0.5	0.5
10	6.2	4.5	5.5
Mean	11.67	5.10	4.45
Median	11.89	3.49	5.00

^A^Distances are given in Haldane cM.
